# A Bibliometrics-Enhanced, PAGER-Compliant Scoping Review of the Literature on Paralympic Powerlifting: Insights for Practices and Future Research

**DOI:** 10.3390/healthcare10112319

**Published:** 2022-11-19

**Authors:** Luca Puce, Khaled Trabelsi, Carlo Trompetto, Laura Mori, Lucio Marinelli, Antonio Currà, Emanuela Faelli, Vittoria Ferrando, Patrick Okwen, Jude Dzevela Kong, Achraf Ammar, Nicola Luigi Bragazzi

**Affiliations:** 1 Department of Neuroscience, Rehabilitation, Ophthalmology, Genetics, Maternal and Child Health (DINOGMI), University of Genoa, 16132 Genoa, Italy; 2 Research Laboratory: Education, Motricity, Sport and Health, EM2S, LR19JS01, University of Sfax, Sfax 3000, Tunisia; 3 High Institute of Sport and Physical Education of Sfax, University of Sfax, Sfax 3000, Tunisia; 4 Istituto di Ricovero e Cura a Carattere Scientifico (IRCCS) Ospedale Policlinico San Martino, 16132 Genova, Italy; 5 Academic Neurology Unit, A. Fiorini Hospital, 04019 Terracina, Italy; 6 Department of Experimental Medicine (DIMES), Section of Human Physiology, University of Genoa, 16132 Genoa, Italy; 7 Centro Polifunzionale di Scienze Motorie, University of Genoa, 16132 Genoa, Italy; 8 Effective Basic Services (eBASE), Bamenda 5175, Cameroon; 9 Laboratory for Industrial and Applied Mathematics (LIAM), Department of Mathematics and Statistics, York University, Toronto, ON M3J 1P3, Canada; 10 Academic Institute of Sport Sciences, Otto-von-Guericke University, 39104 Magdeburg, Germany

**Keywords:** para powerlifting, athletes with disabilities, scoping review, PAGER framework, bibliometrics

## Abstract

Paralympic powerlifting (PP), formerly known as “International Paralympic Committee” (IPC) powerlifting, is the format of powerlifting adapted for athletes with disabilities, and it differs from the version for able-bodied athletes in that it consists of bench press only. According to the mandate of the IPC, PP athletes should be enabled to achieve sporting excellence. As such, rigorous evidence is needed. However, to the best of our knowledge, there exists no systematic assessment of the body of scholarly evidence in the field of PP. Therefore, the present study was conducted to fill in this gap of knowledge, by conducting a scoping review of the literature enhanced by a bibliometrics analysis and by mining two major scholarly databases (MEDLINE via PubMed and Scopus). The aim was to provide a review/summary of the findings to date to help practitioners and athletes. Thirty-seven studies were retained in the present study. These covered the following thematic areas: (i) warm-up strategies (*n* = 2); (ii) aspects of training (*n* = 2); (iii) physiological aspects and responses (*n* = 2); (iv) psychological aspects and responses (*n* = 2); (v) biomechanics of bench press (*n* = 8); (vi) recovery strategy (*n* = 5); (vii) impact of the disability and type of disability (*n* = 4); (viii) epidemiology of PP (*n* = 6); and (ix) new analytical/statistical approaches for kinematics assessments, internal load monitoring, and predictions of mechanical outputs in strength exercises and in PP (*n* = 6). Bibliometrics analysis of the PP-related scientific output revealed that, despite having already become a paralympic sports discipline in 1984, only in the last few years, PP has been attracting a lot of interest from the community of researchers, with the first scholarly contribution dating back to 2012, and with more than one-third of the scientific output being published this year (2022). As such, this scholarly discipline is quite recent and young. Moreover, the community dealing with this topic is poorly interconnected, with most authors contributing to just one article, and with one single author being a hub node of the author network. Distributions of the number of articles and the authors/co-authors were found to be highly asymmetrical, indicating that this research is still in its infancy and has great room as well as great potential to grow. Reflecting this, many research topics are also overlooked and underdeveloped, with the currently available evidence being based on a few studies.

## 1. Introduction

Powerlifting, formerly known as “International Powerlifting Federation” (IPF) powerlifting, is a strength sport in which the maximum possible performance is sought in terms of kilos lifted on a single repetition over three barbell disciplines: namely, back squat (BS), bench press (BP), and deadlift. Each athlete has three attempts for each discipline and must perform at least one successful lift in each of them, otherwise, the athlete does not get a “total” and is disqualified from the competition. The sum or total of the best lift in each discipline determines the winner [1,2,3].

Paralympic powerlifting (PP), formerly known as “International Paralympic Committee” (IPC) powerlifting, is the format adapted for athletes with disabilities, and it differs from the version for able-bodied athletes in that it consists of BP only [4]. Another difference is disability-specific: whilst athletes competing in IPF powerlifting are required to place their feet on the floor, IPC para-athletes execute the lift with their torso, legs, and heels extended over a bench. To make this accessible and safe, the lower center section of the bench is wider than its IPF counterpart and is equipped with straps to stabilize the athlete. Additionally, IPC powerlifting requires an IPC license and appropriate classification status for all athletes. This system does not apply to IPF powerlifting [4]. 

According to the rules of the IPC, the PP discipline is open to male and female athletes aged at least 14 years, characterized by impairments in muscle and joint functions (i.e., strength, or range of motion, ROM), movement deficiencies (athetosis/hypertonia/ataxia), differences in physical structure (lower limb deficiency/amputation, leg length discrepancy, and short stature/dwarfism), and/or a range of physical disabilities (cerebral palsy, spinal cord injury, or poliomyelitis). Moreover, to be eligible, an athlete should be able to fully grip the bar without aids or prostheses, extending the arms with no more than a 20-degree loss of full extension on each elbow joint during the lift. All athletes compete in a single sports class, stratified into ten different weight categories per gender [4], specifically ranging from “49 kg” to “+107 kg” for men, and from “41 kg” to “+86 kg” for women [4]. In this para sport, the athletes can achieve world and Paralympic records equal to or, often, exceeding equivalent able-bodied BP records. Top PP athletes can lift more than three times their body weight. For example, during the Tokyo 2020 Games, a Malaysian male lifter weighing in at the “under 72 kg class” successfully lifted 228 kg. Para-athletes can generally reach these performance-related outcomes between their early- and mid-thirties, after many years of high-intensity daily training (5–6 times per week), and are very similar in terms of load parameters (volume, intensity, and recovery) as their able-bodied counterparts. Besides, para powerlifters dedicate all of their training time to the BP only, differently from able-bodied athletes who also have to dedicate their time to the other two powerlifting disciplines (BS and deadlift).

According to the mandate of the IPC, PP athletes should be enabled to achieve sporting excellence. As such, rigorous evidence is needed to effectively protect and promote PP athletes. This implies the design and implementation of studies aimed at the development and validation of an array of measures and indicators that can monitor and predict performance-related outcomes, the reliability of which has to be tested on large, representative samples [5]. 

However, to the best of our knowledge, there exists no systematic assessment of the body of scholarly evidence in the field of PP. Therefore, the present study was conducted to fill in this gap of knowledge and to provide a review/summary of the findings to date to help practitioners and athletes. 

## 2. Materials and Methods

### 2.1. Study Design and Theoretical Framework 

A literature review is aimed at collecting and appraising the body of evidence from the available scholarly literature, describing the state-of-the-art in terms of the latest advancements, consolidated current knowledge, and gaps in knowledge to address, and guiding future research in the field. Utilizing the “Search, AppraisaL, Synthesis and Analysis” (SALSA) framework, Grant and Booth have identified [6] 14 types of literature reviews based on the research needs, the depth and breadth of the research question(s), and the aims. According to the authors, a scoping review can be defined as a “preliminary assessment of potential size and scope of available research literature” with the “aims to identify nature and extent of research evidence (usually including ongoing research)” [6]. The research question is generally broad, and the researcher’s aims include (i) providing the scholarly community with a (quick and rapid) scoping of the research area(s), (ii) understanding whether the research area(s) is/are worthy of carrying out a more systematic/systematized synthesis approach (i.e., a systematic/systematized review and/or a meta-analysis), (iii) summarizing/synthesizing the literature in terms of major findings, and (iv) identifying critical aspects and gaps in knowledge [6,7].

A scoping review is generally conducted when there exists a significantly heterogeneous body of literature and when no previous systematic review can be detected [8,9]. Here, we leveraged Arksey and O’Malley’s five-step methodology (and subsequent theoretical refinements) [10,11], which consists of (i) identifying the research question(s), (ii) identifying the body of relevant studies, (iii) selecting the studies to include, (iv) charting the data, and (v) collating, summarizing, and reporting the results. 

Moreover, the “Patterns-Advances-Gaps-Evidence for Practice-Recommendations” (PAGER) framework developed by Bradbury-Jones et al. [12] was here exploited. P enables the synthesis of the major findings in terms of unique key themes/thematic areas. A allows scholars to discover the dynamic unfolding of these themes. G enables the identification of under-developed/overlooked themes that should be explored and investigated in future research. E can provide relevant actors and stakeholders (athletes, coaches, instructors) with practical information that can be translated into relevant practices (i.e., training methodologies, conditioning strategies, etc.). Based on G, R can guide future research. 

### 2.2. Research Question(s)

We aimed to summarize the body of existing research on PP by (i) appraising the available evidence, (ii) identifying the existing knowledge and practice shortcomings and gaps, (iii) translating evidence into training recommendations and policies, if possible, and (iv) outlining future prospects and directions in the field. 

### 2.3. Identification of Relevant Studies 

The following keywords were used: “paralympic powerlifting”, “para powerlifting”, “paralympic powerlifter”, and “para powerlifter”. These keywords were properly combined in a search string using the “OR” Boolean connector. Two major electronic, scholarly databases were searched from inception: namely, MEDLINE (via its freely available interface PubMed) and Scopus, without language filters/restrictions. The search was conducted from inception up to 1 October 2022. 

### 2.4. Study Selection and Inclusion/Exclusion Criteria 

Inclusion and exclusion criteria were devised according to the PICOS mnemonic: P (population), paralympic powerlifters; I (intervention), any kind of interventional strategy (warm-up or training/condition program, nutritional supplementation, pharmacological intervention, recovery strategy, etc.); C (comparison/comparator), any kind of comparison (between disabled and able-bodied athletes, the impact of age, sex/gender, weight category, years of experience and training, competing level—regional, national, international—the type of disability/impairment, and if congenital/acquired); O (outcome/outcomes), any outcome relevant to PP (kinematic, biomechanical, physiological, psychological or psychophysiological, epidemiological, methodological, etc.); S (study design), any original study with sufficient details. Reviews were not included but were scanned to increase the chance of getting any relevant study, whilst commentaries, letters to the editor, editorials, expert opinions, or technical notes without sufficient details were discarded. Articles were also excluded if focusing on other paralympic disciplines or reporting data in such a way that it was not possible to disaggregate them and extract data related to PP only. 

### 2.5. Charting the Data 

Data were abstracted utilizing an ad hoc designed and customized Excel spreadsheet. 

### 2.6. Collating, Summarizing, and Reporting the Results 

Abstracted data were presented in a narrative fashion, using tables and figures. Finally, findings were also visualized by means of bibliometrics/scientometrics, which is an emerging, highly specialized branch of information science that allows the rigorous, quantitative assessment of emerging research, in terms of topics, patterns, trends, and hot spots in the scientific literature [13].

### 2.7. Bibliometrics Analysis 

Using VOSviewer version 1.6.18 [14], Gephi [15], and Cytoscape [16], data extracted from MEDLINE via PubMed and RIS reference manager files were mapped and visualized as graphs/networks of scholars (authors/co-authors, known as bibliographic—authorship/co-authorship—graphs/networks) and organizations/institutions. Moreover, the topology of these graphs/networks was investigated in-depth from a quantitative standpoint, by computing a range of several graph theory/network-related indicators, including (i) the number of scholars (authors/co-authors, in the case of a bibliographic graph/network), (ii) the number of countries, (iii) the number of items per author/research organization (both as absolute and relative (%) figures), (iv) the number of connected components (as a proxy for the connectivity of a network), (v) the average number of neighbors, (vi) the number of links, (vii) the total link strength (known also as total edge weight), (viii) the length of paths, such as the shortest paths (known as distance), the average shortest path length (known as the characteristic path length), and other related parameters, (ix) the network diameter and the network radius, (x) the network density, (xi) the network heterogeneity, (xii) the network centralization, (xiii) the number of scholar (author/co-author) clusters (also known as communities), (xiv) the number of research organization/institution clusters/communities, and finally, (xv) the clustering coefficient.

More specifically, graphs/networks were treated, modeled, and analyzed as “undirected networks”. In graph/network theory, undirected networks can be defined as sets of objects (called nodes or vertices) that are connected/linked together, in which all the edges (known also as links) are bidirectional. In undirected networks, two nodes (scholars –authors/–coauthors, organizations, or institutions) are defined as connected if there is a path of edges between them. In addition, between any pair of nodes, there can be no more than one coupling link, even though each link has its own strength, represented by a positive figure, that is assigned in such a way that the higher (lower) this value, the stronger (weaker) the link. The strength of the link may vary, indicating, for instance, the number of quotations shared by two publications, the number of publications two researchers have co-authored, or the number of publications in which two terms/keywords occur together.

Within an undirected graph/network, all nodes that are pairwise connected form a connected component. The number of connected components is an indicator of paramount importance, in that it indicates the connectivity of a network—a lower (higher) number of connected components suggests stronger (weaker) connectivity. The length of a path is computed as the number of edges forming it. There may be multiple paths connecting two given nodes. The shortest path length, also called the distance between two nodes (node *n* and node *m*), is denoted by L(*n,m*). The network diameter is the maximum distance between two nodes. If a network is disconnected, its diameter is the maximum of all diameters of its connected components, whilst the network radius can be defined as the minimum distance between two nodes.

Concerning graph/network paths and path lengths, the average shortest path length, also known as the characteristic path length, gives the expected distance between two connected nodes. Parameters related to the neighborhood include the neighborhood of a given node *n*, which is defined as the set of its neighbors. The connectivity of *n*, denoted by *k_n_*, is the size of its neighborhood. The average number of neighbors indicates the average connectivity of a node in the network. A normalized version of this parameter is known as the network density. The density is a value between 0 and 1. It shows how densely the network is populated with edges (self-loops and duplicated edges are removed and ignored from the computation). A graph/network that contains no edges and solely isolated nodes has a density of 0. In contrast, the density of a clique is 1. The number of isolated nodes may provide insight into how the network density is distributed. 

Another conceptually/theoretically related parameter is known as network centralization [17]. Graphs/networks, the topologies of which resemble a star, have a centralization close to 1, whereas decentralized networks are characterized by having a centralization close to 0. The network heterogeneity as a topological parameter reflects the tendency of a network to contain hub nodes [18]. In addition, the number of multi-edge node pairs indicates how often neighboring nodes are linked by more than one edge. 

Finally, in undirected networks, items can be grouped into non-overlapping clusters, with a cluster being a unique set of items sharing common features included in a map [14]. The clustering coefficient *C_n_* of a node *n* is defined as:

$$C_{n}=\frac{2e_{n}}{k_{n}\left(k_{n}-1\right)}$$

where *k_n_* is the number of neighbors of the node *n*, and *e_n_* is the number of connected pairs between all the neighbors of the node *n*. The clustering coefficient is a ratio (namely, N/M), where N is the number of edges between the neighbors of the given node *n*, and M is the maximum number of edges that could possibly exist between the neighbors of the given node *n*. The clustering coefficient of a node is always a number between 0 and 1. The network clustering coefficient is defined as the average of the clustering coefficients for all nodes in the network. Nodes with less than two neighbors are assumed to have a clustering coefficient of 0.

Finally, the number of papers per year was also visualized as a time series.

## 3. Results

### 3.1. Literature Search 

The initial literature search yielded a pool of 65 items (*n* = 37 from MEDLINE via PubMed and *n* = 28 from Scopus). A total of 21 items were duplicated and were, as such, removed, and 44 items were inspected. Seven studies [19,20,21,22,23,24,25] were excluded with reason (*n* = 2, not reporting sufficient details; *n* = 5, not disaggregating data according to para-sports discipline). Finally, 37 studies [26,27,28,29,30,31,32,33,34,35,36,37,38,39,40,41,42,43,44,45,46,47,48,49,50,51,52,53,54,55,56,57,58,59,60,61,62] were retained in the present scoping review. We found that the included studies focused on a range of aspects involving health, classification, the etiology of injuries, and performance.

### 3.2. Warm-Up Strategies in Paralympic Powerlifting

Two randomized, cross-over studies [26,27] in a sample of 12 elite Brazilian male PP athletes (aged 24.14 ± 6.21 years, body weight 81.67 ± 17.36 kg, experience 4.45 ± 0.31 years) [26] and in a sample of 15 elite Brazilian male PP athletes (aged 28.47 ± 5.79 years, body weight 81.75 ± 17.33 kg, experience 2.43 ± 1.03 years) [27] investigated the impact of three different warm-up conditions (no warm-up, traditional warm-up consisting of dynamic resistance exercises, and stretching warm-up) on a set of PP performance-related outcomes and variables. These included dynamic (1-RM and mean propulsive velocity) and isometric strength (rate of force development, maximum isometric force, time to maximum isometric force, fatigue index, impulse, variability, peak torque) and skin temperature. The authors found no differences among the experimental conditions with the exception of skin temperature over pectoral muscles (overall *p* = 0.038), in particular during the traditional warm-up vs. without a warm-up (*p* = 0.049), whereas the difference between stretching warm-up and without warm-up was borderline significant (*p* = 0.064). Finally, no differences could be detected between traditional and stretching warm-ups (*p* = 0.934). In addition, differences could be computed between the “after” condition without warm-up and stretch warm-up. Without warm-up demonstrated a difference in relation to a traditional warm-up in the “10 min later” condition. Another difference could be described for the maximum isometric force (*p* = 0.005), which was the highest in the without-warm-up condition. Overall, despite these differences, different types of warm-up methods do not seem to influence performance-related outcomes in elite PP athletes.

### 3.3. Aspects of Training in Paralympic Powerlifting

Aidar et al. [28] compared the effect of two different three-week training sessions (elastic bands vs. fixed resistance) conducted in randomized order, through static (maximum isometric force, peak torque, rate of force development, and time to maximum isometric force), dynamic indicators of force (1 repetition maximum, 1RM), and fatigue in a sample of 12 PP athletes (aged 28.60 ± 7.60 years). The authors found an increase in force between pre- and post-training for 1RM (*p* = 0.018, effect size (ES) = 0.412), maximum isometric force (*p* = 0.011, ES = 0.415), peak torque (*p* = 0.012, ES = 0.413), and the rate of force development (*p* = 0.0002, ES = 0.761), suggesting that training with the use of elastic bands has more detrimental effects compared to the method with fixed resistance, promoting overload, increasing fatigue, and decreasing strength. 

Lopes Silva et al. [29] considered 4676 results (1683 achieved by female PP athletes, and 2993 achieved by male PP athletes) in the World Para Powerlifting events (Regional Games/Championships, World Cup, World Championships, and Paralympic Games) between 2014 and 2020, to determine the optimal preparation interval for success. The authors found that there were no significant sex-/gender-specific differences (*p* = 0.37). In addition, no differences could be computed in terms of weight categories (*p* = 0.95). Furthermore, the authors found that the longer intervals corresponded to the most important events. Specifically, the odds of winning a medal at the Paralympic Games were 2.17 (*p* = 0.011) times greater when preparation was ≥40 weeks than when preparation was less than 23 weeks. Considering the World Championships, the odds of winning a medal were 2.34 times greater (*p* = 0.002) when the interval varied from 23 to 31 weeks compared to a preparation interval of <23 weeks. World cup races, on the other hand, are generally career stages that are useful for the athlete to achieve physical fitness or qualification for higher-level competitions. In fact, athletes competing in these events were 1.69 times more likely to win a medal with preparation ranging from 22 to 30 weeks compared with preparation lasting < 11 weeks (*p* = 0.004). Finally, there were no significant differences between the interval of preparation for the Regional Games/Championships.

### 3.4. Physiological Aspects and Responses in Paralympic Powerlifting 

Two studies [30,31] investigated the physiological responses in PP. Paz et al. [30] conducted a randomized cross-over trial to explore post-exercise hypotension after two high-intensity resistance-training sessions in a sample of ten national-level PP athletes (aged 26.1 ± 6.9 years; body weight 76.8 ± 17.4 kg). The authors found a decrease in systolic blood pressure by 5–9% after 90% and 95% of 1RM at 20–50 min post-exercise. Moreover, an increase in myocardial oxygen volume and the double product could be described immediately after and 5 min post-exercise, with the heart rate elevating post-exercise but returning to baseline values by 50 min after training sessions for both training conditions. 

Aidar et al. [31] compared hemodynamic responses (systolic, diastolic, and mean blood pressure, heart rate, heart pressure product, and myocardial oxygen volume) in PP vs. powerlifting before and after training and up to 60 min after training in a sample of 20 athletes. The systolic blood pressure increased after training (*p* < 0.001), and there were differences in the post-training and 30, 40, and 60 min later (*p* = 0.021), between 10 and 40 min after training (*p* = 0.031), and between the two samples (*p* = 0.028), with PP having a stronger and more persistent hypotensive effect, which remained present even after 50 min. Mean blood pressure showed a similar trend, with statistically significant differences between before and after (*p* = 0.016) and 40 min later (*p* = 0.040), and with lower values in PP athletes. Diastolic blood pressure, on the contrary, did not show any difference between powerlifters and PP athletes. Heart rate exhibited differences between before and after, and 5 and 10 min later (*p* = 0.002), and between after and 10, 20, 30, 40, 50, and 60 min later (*p* < 0.001). Heart pressure product and myocardial oxygen volume showed differences between before and after (*p* = 0.006) and between after and 5, 10, 20, 30, 40, 50, and 60 min later (*p* < 0.001). Overall, no risk of hemodynamic overload could be found in PP athletes as well as in their able-bodied counterparts, who exhibited clinically comparable responses to high-intensity resistance training [30,31]. 

### 3.5. Psychological Aspects and Responses in Paralympic Powerlifting 

Only two studies [32,33] explored the psychophysiological responses in PP. Da Silva et al. [32] found that, in a sample of seven male athletes (aged 41.0 ± 10.1 years; body weight 84.7 ± 21.1 kg) undergoing a 4-week program of strength training, the increase in maximum dynamic strength (*p* < 0.001; ES = 0.50) was paralleled by an increase in stress as measured by means of the “Recovery Stress Questionnaire for Athletes” (RESTQ-Sport) scales, with significant increases in the lack of energy (*p* < 0.022; ES = 1.30), success (*p* < 0.035; ES = 0.33), and sleep quality (*p* < 0.007; ES = 0.62). Conversely, there was a post-training decrease in the scores of general well-being (*p* < 0.012; ES = 2.18), interval disturbances (*p* < 0.021; ES = 3.14), personal acceptance, and self-regulation (*p* < 0.006; ES = 2.21). The domains of tension (*p* < 0.003; ES = 1.32), fatigue (*p* < 0.002; ES = 0.72), mental confusion (*p* < 0.002; ES = 2.09), depression (*p* < 0.001; ES = 5.00), and anger (*p* < 0.001; ES = 4.75) reported significantly increased scores. Besides, the vigor domain score was found to be significantly reduced (*p* < 0.001; ES = 0.87). These negative changes in a set of psychophysiological indicators were potentially induced by overload. They can be utilized by coaches to monitor and control the internal training load, ideally customizing the prescription of training loads for PP athletes based on their individual responses. 

The other study [33] is a qualitative case study highlighting the experiences and coping functions of a 35-year-old PP female athlete named Niza, from the socio-cultural context of an Islamic state in Malaysia. The author coupled Foucauldian theory with feminist poststructuralism, narrative inquiry, and a Gestalt phenomenological approach to identify the main discourses embedded within the narrative of the athlete, in an attempt to disentangle the complex dynamics of disability, athleticism, culture, ethnicity, and gender. Several themes emerged, including the initial negative reactions from her family members at the communication of the decision to pursue a career as an athlete, the barriers of society’s conservative and exclusive attitudes toward women, and the lack of encouragement and support. Gradually, Niza was able to challenge this misconception through anticipatory and proactive coping functions, self-consciousness, and strong positive beliefs and became a confident, successful, and inspirational figure for other Muslim female athletes with disabilities.

### 3.6. Biomechanics of Bench Press in Paralympic Powerlifting 

Eight studies [34,35,36,37,38,39,40,41] investigated the biomechanics of BP in PP, including the performance of PP under two different BP conditions (namely, with the legs tied and untied [34,35]), the impact of the choice of a specific grip width [36,37], the effects of arched and flat techniques [38], the impact of a partial vs. full range of movement (ROM) training [39], the evaluation of strength and muscle activation indicators in sticking point region [40], and the force output during different phases of the PP BP movement [41]. 

Guerra et al. [34] analyzed the variations in sEMG (muscle activity of *triceps brachii*—long head, anterior deltoid, and *pectoralis major*—sternal and clavicular portions), the velocity of the barbell displacement (maximum velocity and mean propulsive velocity), and power in the BP at various relative loads (40%, 60%, 80%, and 100% of 1RM) in a sample of 15 PP male athletes (aged 22.27 ± 10.30 years). The authors found no statistical differences in muscle activity in both BP conditions but indicated some intra-individual variability. Specifically, higher muscle activation values were found in the pectoral (sternal portion) than in the anterior deltoid (*p* = 0.035), with a 40% 1RM load in the tied condition. In the untied condition with a load of 60% of 1RM, on the other hand, muscle activation showed higher values in the pectoral (clavicular portion) than in the anterior deltoid (*p* = 0.018) and *triceps brachii* (*p* = 0.046). In the same condition but with a maximum load (100% 1RM), the brachial *triceps* had higher values than the anterior deltoid (*p* = 0.047). Comparing the velocity variables, significant differences were found (*p* < 0.001) between all loads (% 1RM) in both BP conditions, indicating a reduction in velocity due to the increase in the relative load. As for power, similar results were found. However, for the relative load of 40% of 1RM in the untied condition, power was lower than in the 60% and 80% of 1RM. Furthermore, power with a load of 100% of 1 RM differed from all other relative loads (*p* < 0.001) in both BP conditions. In conclusion, the findings showed the predominance of activation of the *pectoralis major* clavicular portion in the tied condition and *pectoralis major* sternal portion in the untied condition in loads of 40% to 60% 1RM, with greater muscle activation of the *triceps* in loads of 100% 1RM. Furthermore, a strong load–velocity relationship and, to a lesser extent, a strong load–power relationship were found. Mota et al. [35] recruited a sample of 16 male PPs, 8 of whom were trained (aged 26.25 ± 6.96 years) and 8 of whom were beginners (aged 30.29 ± 7.34 years), who conducted 40%, 45%, and 50% of 1RM in tied and untied conditions. No differences between those trained and beginners, as well as between the tied and untied conditions, in terms of average propulsive speed and average speed could be found. However, power at 40% of 1RM resulted in significantly higher values for the aforementioned variables in trained PP athletes across both conditions, tied (*p* = 0.033) and untied (*p* = 0.024), since it can be hypothesized that those trained develop more power than beginners. On the other hand, being tied does not create a performance advantage. 

Dos Santos et al. [36,37] conducted two randomized controlled studies consisting of a sample of 15 elite Brazilian male PP athletes (aged 25.40 ± 3.30 years), which aimed at exploring the effects of using different grip widths on BP performance. In the first study, Dos Santos et al. [36] evaluated isometric (time and force spent to reach 30%, 50%, and 100% of the maximal isometric strength) and dynamic (mean propulsive velocity, and force production using 25%, 50%, and 100% of 1RM load) strength. In addition, an electromyographic evaluation was performed during the evaluation of the maximal isometric strength. All evaluations have been carried out with different grip widths in random order (bi-acromial distance: BAD, 1.3 BAD, 1.5 BAD, and 81 cm). Moderate and small effects were described for force production, with 25% (*p* = 0.08), 50% (*p* = 0.41), and 100% (*p* = 0.66) of 1RM load between the grip widths used, respectively. Large and moderate differences were computed between the mean propulsive velocity when performed with different grip widths using 25% (*p* = 0.02), 50% (*p* = 0.15), and 100% (*p* = 0.18) of the maximal dynamic strength load. The highest values for both force generation and mean propulsive velocity were obtained with the 1.5 BAD grip width. Greater lift distances were carried out during BP with 25% (*p* = 0.05) and 50% (*p* = 0.02) of 1RM in BAD conditions. No statistical difference was described in the force values at 30% (*p* = 0.96), 50% (*p* = 0.91), and 100% (*p* = 0.91) of maximal isometric strength between the different grip widths, with the 1.5 BAD grip width condition exhibiting the greatest force generation. Furthermore, statistically significant differences could be computed in time to achieve 30% (*p* = 0.03), 50% (*p* = 0.03), and 100% (*p* = 0.03) of the maximal isometric strength. Finally, sEMG showed moderate, although not statistically significant, effects in terms of muscle activation and the different amplitudes of the grip. 

In the second study, Dos Santos et al. [37] analyzed variables related to the velocity of the barbell displacement (average velocity, average velocity propulsive, and velocity peak) carried out with loads of 30% and 50% of 1 RM with different grip widths in random order (BAD 1.3 × BAD, 1.5 × BAD). The authors found only a significant variable in this study. Specifically, the average velocity was higher with 1 × BAD at 30% of 1RM compared to the 1.3 × BAD. There was also an inverse relationship between load and velocity as the average velocity generated for 50% of the 1RM load was less than that applied for 30% of 1RM. Overall, the findings of these two studies [36,37] indicated the importance of choosing the proper grip width and its impact on muscle activation and performance-related outcomes.

The arched technique (or the arch bridge technique) is when the athlete performs a marked hyperlordosis in the spine, along with scapular retraction. Neto et al. [38] compared the arched and flat techniques in 23 experienced PP athletes vs. 20 beginners. The total load, the trajectory of the barbell in the sagittal plane, and the mean velocity of the barbell in eccentric and concentric phases were computed. No statistically significant differences between the arched and flat techniques for the total load could be found in terms of all analyzed outcomes, with trivial and moderate ESs for experienced and beginner PP athletes, respectively, and with higher values reported for the arched technique in experienced individuals and greater improvements reported for the arched technique in beginner subjects. During the eccentric phase of the BP, all outcome differences presented trivial-to-moderate ESs. The vertical displacement was lower in the arched technique compared with the flat technique for both experienced and beginner athletes, in eccentric and concentric phases. Finally, the root mean square error (RMSE) and the horizontal displacement exhibited nonsignificantly lower values in the arched technique in experienced athletes compared with beginner individuals during the eccentric and concentric phases of the BP. As such, according to this study [38], instead of imposing the arched technique, the most effective technique for experienced and beginner PP athletes should be identified by sports trainers and coaches, based on variables such as the injury level and its characteristics (i.e., structured severe scoliosis or high levels of spinal cord injury).

In training, partial movements are considered a variation of the BP and are generally used to improve control in particular areas of the trajectory or to stimulate the central nervous system without putting stress on it. Mendonça et al. [39] compared the fatigue index, the force production (maximum isometric force, time to maximum isometric force, and rate of strength development), the muscle thickness (clavicular and sternal portions of *pectoralis major*), and the variations in sEMG (muscle activity of the anterior portion of the deltoid muscle, the clavicular portion of the *pectoralis major*, and the sternal portion of the *pectoralis major*) involved in partial vs. full ROM before and after training in a sample of 12 athletes (aged 28.60 ± 7.60 years). In both exercise conditions, time in the rate of force development (*p* < 0.001, ES = 0.720) and time in the rate of strength development (*p* = 0.014, ES = 0.437) exhibited decreased values post-training. Moreover, the maximal isometric force decreased in post-training as well to a greater extent in full ROM (*p* < 0.001, ES = 3.53) than in partial ROM (*p* < 0.001; ES = 1.85), while the fatigue index increased solely in the partial ROM (*p* < 0.001; ES = 1.65). Regarding the other variables, the clavicular portion of the *pectoralis major* muscle thickness from pre- to post-training increased more in full ROM (*p* < 0.001; ES = 3.33) than in partial ROM (*p* < 0.001; ES = 2.34). Further, similar increases were found in the sternal portion of the *pectoralis major* muscle thickness between full ROM (*p* < 0.001; ES = 1.71) and partial ROM (*p* < 0.001; ES = 2.36). Finally, both portions of the *pectoralis major* were more active in full ROM (*p* < 0.05), while the *triceps* muscle was more active with partial ROM (*p* < 0.05). In conclusion, compared to a full lift, partial ROM training allows the management of higher workloads with fewer losses in muscle functions.

The concentric phase in the BP exercise is conventionally divided into three different regions: (i) pre-sticking: time from the lowest point of the bar to the maximum velocity of the bar, (ii) sticking: from the maximum velocity of the bar to the first minimum velocity of the bar, and (iii) post-sticking: from the moment the acceleration of the bar has returned positive up to the second peak of maximal velocity.

Aidar et al. [40], in a sample of 12 PP athletes (aged 26.56 ± 5.55 years), evaluated changes in strength indicators (maximum isometric force, rate of force development, and time to maximum isometric force), kinematic parameters (velocity and dynamics time), and sEMG muscle activity (pectoral, sternal and clavicular parts, deltoid and *triceps*) at different distances from the bar to the chest (5.0; 10.0; 15.0 and 25 cm). Furthermore, the velocity and dynamic time in the eccentric and concentric phases (pre-sticking, sticking, and post-sticking) were assessed. The authors found changes in velocity at the various points in the sticking region. Specifically, at 5.0 cm, velocity reaches its highest value (0.699 m/s), whilst at 10.0 cm, it tends to fall (0.198 m/s) (*p* < 0.001), and then increases at 15 cm (0.423 m/s) (*p* < 0.04) and at 25.0 cm (1.137 m/s) (*p* < 0.001). There were also differences in velocity between the pre-sticking region and the sticking region (1.98 ± 0.32 vs. 1.30 ± 0.43, *p* = 0.039) and in the dynamic time between the pre-sticking and the sticking region (0.40 ± 0.16 vs. 0.97 ± 0.37, *p* = 0.021). Regarding the strength indicators, the maximum isometric force showed an increase after the sticking point (10 cm) with significant differences between 5.0 and 15.0 cm (*p* = 0.001), 5.0 and 25.0 cm (*p* < 0.001), and 10.0 and 15.0 cm (*p* = 0.012). The rate of force development was higher at 25.0 cm than at 5 cm (*p* = 0.004) and 10.0 cm (*p* < 0.001). Finally, in the time to maximum isometric force, there were differences between 5.0 cm and 15.0 cm (*p* < 0.001), 5.0 cm and 25.0 cm (*p* = 0.001), 10.0 cm and 15.0 cm (*p* < 0.05), and 15.0 cm and 25.0 cm (*p* < 0.05). The electromyographic results did not indicate significant differences between the muscles and between the different distances studied. However, greater activation of the brachial *triceps* was found compared to the other muscles, mainly at 10.0 cm and 15.0 cm. In conclusion, in the sticking region, the strength and kinematic parameters tend to be altered despite the greater contribution of the *triceps* muscle. These findings have practical implications in that coaches should determine the sticking point and focus on it, devising proper and effective training and conditioning strategies for the point at which the failure occurs. This is anticipated to significantly improve PP outcomes.

Da Silva et al. [41] recruited six male (aged 26.5 ± 8.0 years) and four female (aged 39.8 ± 11.2 years) PP athletes who underwent 1 repetition at 95% intensity of 1RM three times with 5 min of rest between attempts. Electromyographic variables (root mean square (RMS), mean frequency, and median frequency) and kinematics (velocity of movement of the barbell) were evaluated in different sub-phases of the BP movement (sub-phases I and II for the eccentric phase and pre-sticking, sticking, and post-sticking for the concentric phase). There was no significant difference between the total velocity values of the eccentric and concentric phases. However, the eccentric phase was shorter than the concentric one. In the eccentric phase, differences in velocity were found between sub-phases I and II (149.36 ± 53.39 vs. 181.97 ± 47.01). In the concentric phase, on the other hand, the barbell velocity decreased during the sticking sub-phase compared to the pre-sticking phase (122.95 ± 35.92 vs. 179.39 ± 54.68), and the velocity increased again in the post-sticking phase (160.36 ± 65.09). Finally, the barbell velocity in sub-phase II and pre-sticking was significantly higher than in the sticking phase (*p* < 0.05). Regarding the electromyographic results, the RMS values obtained for the *triceps* were significantly lower than those of the pectoral and deltoid muscles for all the sub-phases studied (*p* < 0.05). Except for sub-phase I, where there were no differences in muscle activation, the deltoid had the maximum RMS values for all sub-phases (*p* < 0.05). The behavior common to all the muscles studied was that they had their maximum activation in the pre-sticking phase. In the mean and median frequency, on the other hand, the *triceps brachii* showed the highest values, followed by the deltoid and pectoral muscles. Furthermore, in the *triceps brachii*, statistically different values were found in all the movement sub-phases (*p* < 0.05). In both frequency parameters, all muscles showed significant differences in the post-sticking phase (*p* < 0.05). These results have practical implications in that sports trainers and coaches should develop resistance-training programs in such a way as to include variations in the BP execution and optimize PP performance-related outcomes. 

### 3.7. Recovery Strategy in Paralympic Powerlifting 

Five studies [42,43,44,45,46] explored the impact of post-exercise recovery in PP through physiological and biochemical assessments using different strategies. Aidar et al. [42] conducted a randomized, placebo-controlled trial and recruited 10 PP athletes at the national level, aged 27.13 ± 5.57 years. They underwent a warm-up and 5 × 5 at 80–90% of 1RM, ingesting ibuprofen 15 min before and 5 h after training. Ibuprofen ingestion resulted in positive effects, with greater peak torque values (*p* = 0.04, at 24 h) and a lower fatigue index (*p* = 0.01, at 24 h), even though there was no impact on oxidative stress markers. Blood indicators, including leukocytes, with the use of ibuprofen were higher than with the placebo (*p* < 0.001). 

In another work, Aidar et al. [43] conducted a randomized, placebo-controlled trial and recruited a sample of 20 PP athletes (10 at the national level, aged 32.50 ± 3 years, and 10 at the regional level, aged 30.75 ± 5.32 years). Athletes underwent a warm-up and 5 × 5 at 80% of 1RM, with half of the sample ingesting ibuprofen 15 min before the commencement of the training. Ibuprofen ingestion resulted in greater peak torque values (*p* = 0.007) and a lower fatigue index (*p* = 0.002) in the national level group. Leukocytes, with the use of ibuprofen in the national level group, were greater than in the regional level group (*p* = 0.001). Similarly, neutrophils levels in the national-level group treated with ibuprofen were greater than those in the regional-level group treated with ibuprofen and a placebo (*p* = 0.025). Lymphocytes levels in the national-level group treated with ibuprofen were lower than those in the regional-level group treated with ibuprofen and a placebo (*p* = 0.001). Monocytes levels in the national level group with ibuprofen and a placebo were lower than those in the regional level with ibuprofen (*p* = 0.049). Hemoglobin, hematocrit, and erythrocyte values were higher at the national level with ibuprofen and the placebo than those at the regional level with ibuprofen and a placebo (*p*-value < 0.05). Ammonia levels were higher in the national level group with ibuprofen (*p* = 0.007) and a placebo (*p* = 0.038), with respect to the regional-level group with ibuprofen and a placebo, respectively. 

Fraga et al. [44] recruited eight PP athletes (aged 27.0 ± 5.3 years) competing at the national level, who underwent a warm-up and 5 × 5 at 85–90% of 1RM. Ingestion of ibuprofen or a placebo occurred 15 min before and 5 h after training. The maximal isometric force only decreased in the placebo condition, with a significant increase between 24 and 48 h in the ibuprofen condition, whilst the post-exercise rate of force development decreased significantly for both conditions. Muscle temperature decreased significantly at 48 h post-exercise in the placebo condition, while deltoid muscle temperature at 48 h post-exercise was higher in the ibuprofen condition. Finally, creatine kinase levels were higher with the placebo than with ibuprofen 48 h after exercise, whilst alanine aminotransferase levels were lower 24 h after the training, with ibuprofen. Immediately after training, aspartate aminotransferase levels increase with the placebo, while with ibuprofen, they increase after 24 h. These findings, taken together, seem to indicate that the ingestion of ibuprofen exerts positive effects, in that it appears able to counteract, reduce, or at least partially delay the increases in the levels of creatine kinase and alanine/aspartate aminotransferases—an increase partly induced by the exercise and partly by the underlying disability in this population.

Sampaio et al. [45] investigated the impact of creatine supplementation on the recovery in a sample of 8 PP athletes aged 25.40 ± 3.30 years, undergoing training consisting of 5 × 5 at 85–90% of 1RM. There was no significant variation in the peak torque, rate of force development, time to maximum isometric force, and force with creatine supplementation, whilst the fatigue index after 7 days decreased (*p* = 0.02), making this supplementation a viable nutritional strategy for PP athletes. 

dos Santos et al. [46] investigated how different post-workout recovery strategies (passive recovery, dry needling, and cold-water immersion) can impact homeostasis in a sample of twelve male PP athletes (aged 25.4 ± 3.3 years) undergoing strength training consisting of 5 × 5 at 120% of 1RM in the eccentric phase and 80% of 1RM in the concentric phase + 3 × 5 at 40% of 1RM. The maximal isometric force differed significantly among the three post-workout recovery strategies (*p* = 0.046). In particular, with passive recovery and dry needling, the maximal isometric force was found to decrease compared with the pretest value at 15 min and 2 h. Similarly, in cold-water immersion, it increased from 2 to 24 h and reached 20% more force after 24 h than at the baseline level. Biochemical blood indicators differed as well among the three post-workout recovery methods (*p* = 0.006). In more detail, cold-water immersion and dry needling led to increased levels of interleukin-2 (IL-2) from 24 to 48 h compared to that from 2 h to 24 h. On the other hand, interleukin-4 (IL-4) and interferon-gamma (IFN-γ) levels did not change significantly over time. These molecules, with the exception of IL-4, have a pro-inflammatory activity, which may be detrimental, but if finely regulated and controlled, they can play a key role in muscle repair and regeneration. Muscle thickness was another variable that differed according to the type of recovery strategy (*p* = 0.002). More specifically, with passive recovery, it increased and remained elevated, whilst with cryotherapy, it increased after 15 min and 2 h, whilst after dry needling, muscle thickness did not increase in any of the muscles analyzed, and after 2 h, muscle thickness was found to significantly decrease again in the *major pectoralis* muscle. Finally, pain pressure differed based on the post-workout recovery strategy in a muscle-specific way: differences could be described for acromial *pectoralis* (*p* = 0.003), but not for the deltoid muscle (*p* = 0.085). The pain pressure threshold was found to increase significantly immediately after all recovery methods (15 min). Then, it decreased for all muscles, with the lowest measurement computed 24 h after passive recovery, after which it started increasing again. A similar trend could be found for dry needling, even though the decrease was lower. Finally, after cold-water immersion, pain pressure stabilized after 15 min and increased after 2 h for acromial *pectoralis*. In conclusion, the various recovery strategies had differential effects in terms of the return to homeostasis in PP athletes, impacting edema, pain, and local and systemic recovery to varying degrees and with different, precise timing, with the dry needling method being effective in shorter-term recoveries, and with cold-water immersion being effective in shorter and longer recoveries.

### 3.8. Impact of the Disability and Type of Disability in Paralympic Powerlifting 

Four studies [47,48,49,50] explored the impact of the disability and the type of disability in PP. Gołaś et al. [47] compared two elite flat BP athletes—an elite able-bodied athlete (aged 34 years, body weight 103 kg) and an athlete with a lower limb disability (aged 31 years, body weight 92 kg)—in terms of the activity of four muscles (*pectoralis major*, anterior deltoid, lateral and long heads of the *triceps brachii*). The peak activity of all the considered muscles significantly differed between the two athletes (*p* = 0.001, *p* = 0.001, *p* = 0.0021, and *p* = 0.002, respectively). Differences depended on the load: 60% to 100% 1RM (*p* = 0.007), 70% to 100% 1RM (*p* = 0.016), and 80% to 100% 1RM (*p* = 0.032). These findings can be explained by considering that keeping the feet on the bench leads to an increased engagement of upper body muscles and to their greater activation. 

Szafraniec et al. [48] quantitatively assessed the impacts of a 6-week high-velocity strength training program on movement velocity and strength endurance measured one week before and one week after in eleven experienced powerlifting athletes with cerebral palsy vs. seven control subjects. While movement velocity increased in the cerebral palsy group only (*p* = 0.016), strength endurance increased in both groups (*p* < 0.001 and *p* = 0.049, respectively).

Teles et al. [49] compared PP athletes with (aged 30.57 ± 4.20 years) and without (aged 25.67 ± 4.52 years) spinal cord injuries in terms of the impact of dynamic (mean propulsive velocity, maximum velocity, and power) and static (maximum isometric force, time to maximum isometric force, rate of force development, impulse, variability and fatigue index) force and associated parameters at different intensities on performance-related outcomes. The two groups differed in terms of dynamic (*p* < 0.05) but not static force indicators. Concerning EMG, individuals with injured spinal cords exhibited differences between the *triceps* in relation to the previous deltoid (*p* = 0.012).

Aidar et al. [50] compared 10 PP athletes with spinal cord injuries (aged 30.00 ± 4.27 years) and 10 with other disabilities (aged 28.30 ± 4.92 years) in terms of the impact of a dynamic force (mean propulsive velocity, maximum velocity, and power), with loads of 40%, 60%, and 80% of 1RM in tied and untied conditions Athletes were also assessed in terms of static force (maximum isometric force, time to maximum isometric force, rate of force development, impulse, variability, and fatigue index). The authors found no differences between spinal cord injuries vs. other disabilities in dynamic and isometric strength indicators. However, spinal cord injuries at 80% of 1RM showed a higher mean propulsive velocity in the untied than in the tied condition (*p* = 0.041). Similarly, at 40% (*p* = 0.004) and 80% (*p* = 0.023) of 1RM, spinal cord injuries had a higher maximum velocity in the untied than in the tied condition. These studies [47,48,49,50], taken together, show that PP is a viable strategy in people living with disabilities, in that persons with injuries, such as cerebral palsy or spinal cord dysfunction, still have adequate neuromuscular control after proper training. 

### 3.9. Epidemiology of Paralympic Powerlifting 

Six studies [51,52,53,54,55,56] explored the epidemiological aspects of PP.

Lopes Silva et al. [51] retrospectively assessed 3107 athletes (1985 males and 1122 females) who took part in the last eight World Championships and six Paralympic Games in terms of sex/gender, chronological age, weight category, and competition achievements. The authors found that male athletes were older (33.2 ± 8.6 vs. 32.2 ± 7.5 years, *p* = 0.001, ES = 1.21, large) and stronger. Regarding the age of male athletes, there was a main effect of events (*p* = 0.018, ES = 0.001, small), even though no differences between those competing at Paralympic Games and World Championships (*p* = 0.098) could be found. Moreover, there was a main effect of competition achievement (*p* = 0.001, ES 0.009, small), with medalists being younger compared to non-medalists. Further, there was a main effect of the weight category (*p* = 0.001, ES = 0.014, small). Considering the age of female athletes, there was only a main effect for competition achievement (*p* = 0.001, ES 0.017, small), with medalists being younger when compared to non-medalists (*p* = 0.001), and a main effect for weight category (*p* = 0.001, ES = 0.031, moderate). Male athletes were able to lift heavier loads than females (168.0 ± 35.9 vs. 96.8 ± 24.4 kg, *p* < 0.001, ES = 2.20, large). A similar trend was reported for the relative load (2.06 ± 0.90 kg/body mass (BM) vs. 1.49 ± 0.61 kg/BM, *p* < 0.001, ES 0.71, small). The main effects of competition (*p* = 0.001, ES = 0.017, small), with higher values in the Paralympic Games compared to the World Championships, and of weight category (*p* = 0.001, ES = 0.215, moderate), were computed. Finally, an event and weight category interaction (*p* = 0.045, ES = 0.00, small) was found; for relative load, there was an event and ranking interaction (*p* = 0.046, ES = 0.002, small). Concerning the absolute load of female athletes, there was a main effect of events (*p* = 0.001, ES = 0.024, small), with higher values in Paralympic Games compared to World Championships. Furthermore, there was a main effect from the weight category (*p* = 0.001, ES = 0.344, large), also in terms of the relative load (*p* = 0.001, ES 0.124, large). Finally, in males, chronological age and body mass significantly correlated with the absolute and relative load, whilst in females, age was associated with the relative load as well as body mass with the absolute and relative load. In conclusion, the performances of both groups were better at the Paralympics than at the world championships. The medalists were younger. The lighter weight categories included participants of a younger age and with a greater relative load than the heavier competitors. 

Willick et al. [52] analyzed the injury incidence rate and the injury incidence proportion in PP athletes during the London 2012 Paralympic Games (7 days). Out of 163 athletes participating in the competition, 38 injuries were reported by 38 different athletes. The injury incidence rate and the injury incidence proportion were 33.3 and 23.3, respectively. Lighter weight classes had fewer injuries than heavier weight classes. No significant differences between male and female athletes or age-specific effects could be found. In terms of the onset of injury, chronic-overuse injuries were the most frequent, followed by acute-on-chronic and acute traumatic injuries. Concerning the anatomical location of injuries, the shoulder/clavicle was the most injured area, followed by the chest and elbow. 

Jarraya et al. [53] computed the injury frequency in PP athletes undergoing imaging (X-rays, ultrasound, and magnetic resonance imaging (MRI)) during the Rio 2016 Summer Paralympic Games. Of the 182 athletes participating in the competition, 20 underwent imaging. Of the 33 examinations performed, 18 injuries were reported affecting the upper extremities. The injuries mainly involved the tendons followed by the muscles and bone bruises. 

Hamid et al. [54] determined the sociodemographic, clinical, and anthropometric physical parameters of Malaysian PP athletes during a Powerlifting Workshop and National Championship. Fifty-two athletes representing 13 different Malaysian states were recruited. Most of the participants were men (82.7%), and the mean age was about twenty years (24.50 ± 8.25 years). A spinal cord injury and lower limb amputation were the most frequent pathologies, with a percentage of 34.6% and 26.9%, respectively. About half of the powerlifters (42.3%) competed in international competitions, more than half (76.9%) had at least 1 year or more of experience, a minority (5.8%) also practiced other sports (athletics, basketball, and wheelchair tennis), and nearly all (97%) have completed basic education. The workouts generally had a frequency of two to four sessions per week with a duration of 90 min per session. Regarding the anthropometric characteristics, the authors found that women had a lower lean body mass (54.90, interquartile range (IQR) 14.32 kg, vs. 43.50, IQR 9.78 kg, *p* = 0.031) and higher percentages (19.80 ± 10.56% vs. 35.61 ± 6.08%, *p* ≤ 0.001) and a greater amount of body fat (14.10 ± 11.55 kg vs. 26.50 ± 10.80 kg, *p* = 0.003) than men. Furthermore, males had significantly longer arm and forearm lengths compared with females (30.10, IQR 3.00 cm vs. 23.00, IQR2.13 cm; *p* = 0.020). In the analyses based on weight categories, body mass index (BMI) was significantly highest (*p* < 0.001) among the heavyweight class (42.08 ± 11.39 kg/m^2^) followed by the middleweight (31.33 ± 5.46 kg/m^2^) and lightweight (25.55 ± 7.05 kg/m^2^) classses. The lean body mass among the lightweight, middleweight, and heavyweight classes was 45.90 ± 9.13 kg, 63.41 ± 8.29 kg, and 71.70 ± 10.16 kg, respectively, with significant differences between the middleweight and heavyweight (*p* < 0.001) and between the lightweight and middleweight (*p*-value < 0.001) classes. Significant differences could also be found in the body fat between the lightweight and heavyweight (30.71 ± 12.31 kg vs. 9.88 ± 6.04 kg, *p* < 0.001) classes and between the lightweight and middleweight (9.88 ± 6.04 vs. 17.41 ± 8.40, *p* < 0.001) classes. Similarly, in hip circumference, significant differences were found between the lightweight and middleweight (81.98 ± 20.51 cm vs. 108.72 ± 7.62 cm, *p* < 0.001) classes and between the middleweight and heavyweight (108.72 ± 7.62 vs. 118.38 ± 8.03, *p* < 0.001) classes. Both dominant and non-dominant arm girths during relaxation and tension were significantly greater in the heavier weight classes (*p*-value < 0.001). Finally, variables were found that have a significant correlation with the powerlifters’ best lift. Specifically, strongly correlated variables were arm girth (r ranging from 0.549 to 0.694, *p*-value < 0.0001) and experience (r = 0.724, *p*-value < 0.0001). Weight and BMI, on the other hand, showed moderate correlations (r = 0.418, *p* = 0.009 and r = 0.462, *p* = 0.008), while lean body mass and age only showed weak correlations (r = 0.389, *p* = 0.019 and r = 0.352, *p* = 0.030). 

van den Hoek et al. [55] compared world BP records of different weight classes in terms of absolute and relative strength (strength-to-weight ratio [kg∙kg_bw_^−1^]) between powerlifters with disabilities and powerlifters without disabilities. Surprisingly, the authors found similar results between the two athlete populations and, in some cases, higher world records for powerlifters with disabilities than their counterparts. Specifically, powerlifting world record holders without disabilities showed an absolute strength greater than those with a disability in 5 of 8 weight classes for women (47 kg, 52 kg, 57 kg, 76 kg, and 84 kg) and 6 of 8 weight classes for men (59 kg, 66 kg, 74 kg, 83 kg, 93 kg and +120 kg). Regarding the relative strength, the values ranged, respectively, from 1.83 to 3.88 kg∙kg_bw_^−1^ for powerlifters with disabilities and from 1.49 to 3.35 kg∙kg_bw_^−1^ for powerlifters without disabilities (*p* = 0.118). For women, on the other hand, relative strength values ranged from 1.19–2.72 kg∙kg_bw_^−1^ for powerlifters with disabilities and from 1.14–2.22 kg∙kg_bw_^−1^ for powerlifters without disabilities, respectively (*p* = 0.432). Finally, among powerlifters with disabilities, the greatest relative strength was observed in the 49 kg weight class for males (3.88 kg∙kg_bw_^−1^) and in the 50 kg weight class for women (2.72 kg∙kg_bw_^−1^). Among powerlifters without disabilities, on the other hand, the greatest relative strength was observed in the under-66-kg weight class for males (3.35 kg∙kg_bw_^−1^) and in the under-63-kg weight class for females (2.29 kg∙kg_bw_^−1^). 

Severin et al. [56] conducted a retrospective study with a dual aim: (1) to determine the average age of and weight lifted by 2079 athletes who participated in the Paralympic Games and World Championships, stratified by gender and body weight category and (2) to establish the age-related trajectory of the performance and derive estimates of the age at peak performance. Regarding the first aim, the authors found that the mean age for men and women in the heaviest body weight categories was 36 and 43 years, respectively. In addition, the average age of athletes in the heavier bodyweight categories was higher than that of athletes in the lighter bodyweight categories (*p* < 0.001). Particularly in men, age increases between 49 and 65 kg (3.9 ± 0.1 years, *p* = 0.002), 65 and 80 kg (2.5 ± 0.8 years, *p* = 0.03), and for the body weight categories 80 kg and >107 kg (3.3 ± 1.1 years, *p* = 0.07). For women, on the other hand, there were age increases ranging from 41 to 50 kg (4.4 ± 1.0 years, *p* < 0.001), 50 and 67 kg (5.6 ± 1.0 years, *p* < 0.001), 67 and 86 kg (3.6 ± 1.0, *p* = 0.02), and for body weight categories 86 and >86 kg (4.6 ± 1.1 years, *p* < 0.001). For the second aim, it was found that peak performance in men occurs at a younger age than in women (36.3 ± 0.5 years vs. 40.5 ± 0.7 years, *p* < 0.001). Furthermore, higher-level powerlifters achieved their peak performance earlier than their lower-level peers (37.1 ± 0.7 years vs. 39.7 ± 0.5 years, *p* = 0.003). Finally, the age at which athletes were most likely to reach their full potential (between 31 and 35 years of age) was lower than that measured (36 years for males and 41 years for females) using individual age-related trajectories.

### 3.10. New Analytical/Statistical Approaches for Kinematics Assessments, Internal Load Monitoring, and Predictions of Mechanical Outputs in Strength Exercises and in Paralympic Powerlifting 

Six studies [57,58,59,60,61,62] developed new analytical/statistical approaches for kinematics assessments and predictions of mechanical outputs in strength exercises. The Functional ANOVA (FANOVA) model is a generalized regression model, with logistic, probit, and Poisson regression as special cases. FANOVA enables the modeling of multivariate predictor functions as specified sums of constant terms, main effects, and interaction terms. Ramos Dalla Bernardina et al. [57] recruited eight male and two female PP athletes aged 35.00 ± 7.01 years undergoing a set of five repetitions at intensities of 50% and 90% of 1RM. Mean velocity in the concentric and eccentric phases was assessed using ANOVA and FANOVA approaches to quantify asymmetries at different submaximal intensities In both analyses, a higher average velocity was found at an intensity of 50% compared to 90% of 1RM, specifically for the range of movement from 70% to 100% (*p* = 0.005). Whilst the two methodologies yielded similar results for the eccentric phase (no interaction between limb and intensity, and no main effect of limb, *p* = 0.801), the FANOVA approach enabled the authors to find an asymmetrical pattern in the concentric phase, in favor of the preferred limb, at the maximum intensity of the exercise. ANOVA failed to capture a significant interaction between limb and intensity (*p* = 0.999), as well as the main effect of asymmetry (*p* = 0.526), while the main effect of intensity could be found (*p* = 0.001), identifying higher mean velocities at an intensity of 50% compared to that of 90% of 1RM. When compared with ANOVA, FANOVA analyzes the entire profile of the velocity curve in the concentric and eccentric phases, allowing for a better understanding of the biomechanical characteristics of the movement with respect to the classical approach.

Bellitto et al. [58] conducted a case series analysis of one able-bodied athlete (male, 22 years) andthree PP athletes (one female and two female individuals, aged 20–40 years), undergoing three repetitions at intensities of 90% of 1RM with 3 min of rest. By means of kinematics, the authors evaluated the movements in the sagittal, frontal, and transverse planes. sEMG showed that PP athletes had high symmetry and low variability in the three movements of the bench. The able-bodied athlete had a lower level of repeatability and symmetry. Techniques of execution and muscle patterns were different for each athlete. The instrumental evaluation used allowed for the identification of similar kinematic performance patterns and specific muscle strategies for each athlete. 

Loturco et al. [59] analyzed the relationship between load and barbell velocity (average velocity, average propulsive velocity, and peak velocity) to accurately predict distinct loading intensities (%1RM) during maximum contraction in a sample of eight males (aged 28.3 ± 3.6 years), five females (aged 25.4 ± 5.2 years), and four dwarfs (aged 29.4 ± 3.6 years). Associations between bar velocities and %1RM were strong for all the loading intensities (R^2^ values ranging from 0.80–0.91), but the precision of the predictive modeling equations was lower (~5%) and higher (~20%) at lighter and heavier loading intensities (≤70% 1RM, and ≥70%1RM), respectively. Finally, very strong athletes perform BP 1RM assessments at lower velocities than reported in the scholarly literature. 

Aidar et al. [60] assessed different methodologies (two-, and four-point methods with distal loads, and six-point methods with proximal loads) to evaluate BP maximum repetitions and their impacts on the measurement of the minimum velocity limit, load at zero velocity, and force velocity (FV) in a sample of 15 elite male PP athletes (aged 27.7 ± 5.7 years). The authors found that all methods exhibited a good ability to predict BP 1RM in PP.

Aidar et al. [61] compared dynamic (mean propulsive velocity, maximum velocity, power, and prediction of one maximum repeat) and static (maximum isometric strength, time to maximum isometric strength, rate of force development, impulse, variability, and fatigue index) indicators at different intensities in 11 national level PP athletes (aged 26.13 ± 7.22 years) vs. 12 regional level PP athletes (aged 29.25 ± 4.50 years). The authors found higher velocity values in PP at the regional level compared with the national level. Notably, there were differences for the mean propulsive velocity at 45% (*p* = 0.041), 55% (*p* = 0.047), and 75% (*p* = 0.020) of 1RM and for the maximum velocity at 50% (*p* = 0.041), 55% (*p* = 0.0049), 75% (*p* = 0.013), and 95% (*p* = 0.040) of 1RM. However, the national level has developed higher power rates than the regional level at 40% (*p* = 0.004), 45% (*p* = 0.004), 50% (*p* = 0.023), and 60% (*p* = 0.032) of 1RM and the maximum of the predicted repetition. Regarding static indicators, the national level developed a higher maximum isometric force (*p* = 0.001), impulse (*p* = 0.001) and variability (*p* = 0.049), whilst there were no differences in the time to maximum isometric strength (*p* = 0.262), rate of force development (*p* = 0.276), and fatigue index (*p* = 0.180). In conclusion, national-level athletes rely more on strength than velocity. 

While the “Rating of Perceived Exertion” (RPE) scale can be used for internal load monitoring in athletes undergoing aerobic training, and it has been demonstrated to not be a useful and clinically meaningful tool to capture the strength training load intensity, a possible alternative in PP is given by a scale based on the repetitions in reserve (RIR), which examines how many repetitions the athlete estimates they can perform after the end of the set. Based on this perception, the athlete stipulates a score from 1–10 (1 corresponds to little-to-no effort and 10 to maximum effort). Specifically, the RIR-based scale values are 10, 9.5, 9, 8.5, 8, 7.5, 7, 5–6, 3–4, and 1–2 and are associated with 0, 0.5, 1, 1, 5, 2, 2.5, 3, 5, 6, and 7 repetitions (repetition estimate), respectively. Neto et al. [62] validated the RIR-based scale in a sample of 20 PP. In this study, participants were asked to perform a minimum of one repetition and up to a maximum of 4 for each different intensity (100%, 90%, 85%, 80%, and 75% of 1RM), and after each test, the RIR-based scale was evaluated. Subsequently, the number of repetitions performed was added to the estimate of repetitions provided by the RIR-based scale (estimate of total repetitions). Finally, the estimated total repetitions were compared with a maximal strength test (1RM) and a maximum repetitions test at different intensities (90, 85, 80, and 75% of 1RM). There were no significant differences between the repetitions of the maximum strength test and the estimated total repetition. Similarly, no significant differences were found between the estimated total repetition and the repetitions of the maximum strength test for 100%, 90%, 85%, and 80% of 1RM. However, repetitions performed at 75% of 1RM were significantly higher than the total estimated repetitions (median = 9.0 vs. 7.0 repetitions, ∆ = 13.8%, SE = 0.4). To demonstrate the reliability of the scale, the correlation of the estimated total repetitions with the repetitions of the maximum strength tests for all load intensities was very high (intraclass correlation coefficient (ICC) = 0.91, *p*-value < 0.01). Using the Bland and Altman method, the difference between means was 0.9 repetitions, and the interval around differences was 6.4 repetitions. In terms of construct validity, the RIR-based scale exhibited a high correlation value with 1RM intensities (rho = 0.86, *p*-value < 0.05).

### 3.11. Bibliometrics-Based Analysis of Paralympic Powerlifting Scientific Output 

**Figure 1:**
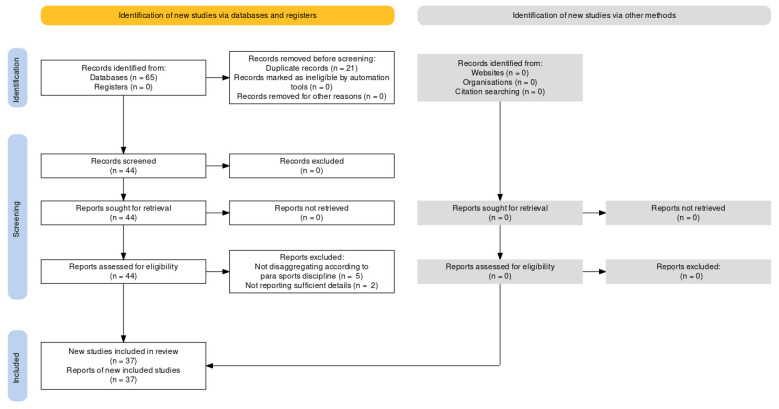
Flowchart adopted in the present scoping review.

**Figure 2:**
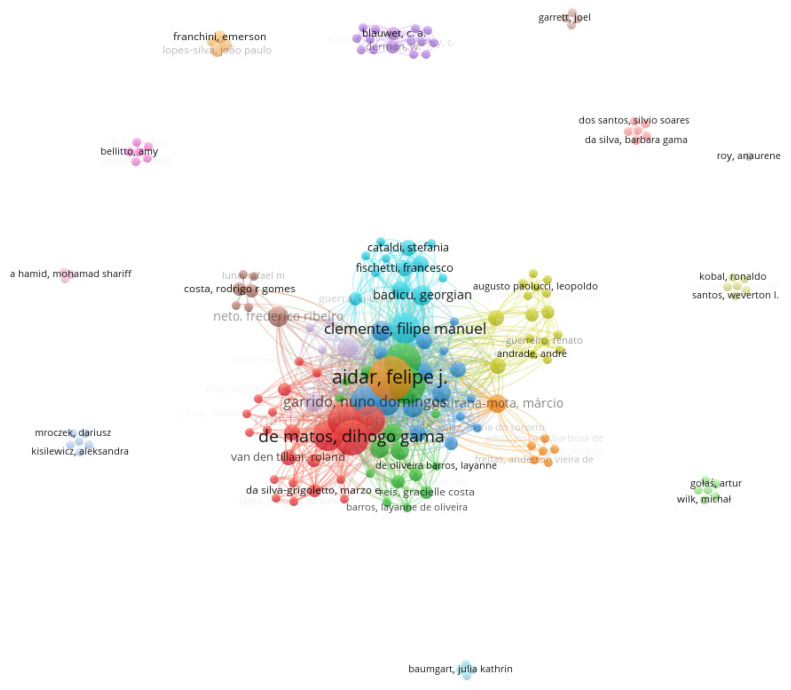
Bibliographic network showing connections between authors active in the field of Paralympic powerlifting.

Our bibliometrics analysis enabled us to identify 164 researchers (nodes), 103 (62.8%) of whom were interconnected (Figure 1). The resulting graph (Figure 2) consisted of 1229 links (edges), with a total link strength of 2065, and 19 clusters. The most prolific author (topologically speaking, the hub node) was Aidar, F. J., with 22 items/documents (representing 59.5% of the scientific output overviewed in the present scoping review). The list of the ten most productive scholars can be found in Table 1.

**Figure 3:**
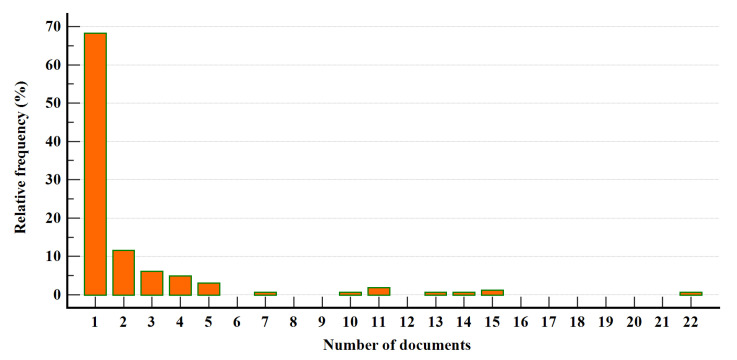
Distribution of Paralympic powerlifting-related documents broken down to authors.

As can be seen from Figure 3, the distribution of documents is highly asymmetrical (median = 1, coefficient of skewness = 3.76, *p* < 0.0001, coefficient of kurtosis = 15.92, *p* < 0.0001). A total of 68.3% of scholars have authored one document, whilst one single author is responsible for about 60% of the PP-related scientific production. The main topological features of the scholarly community of authors on PP are shown in Table 2. 

**Figure 4:**
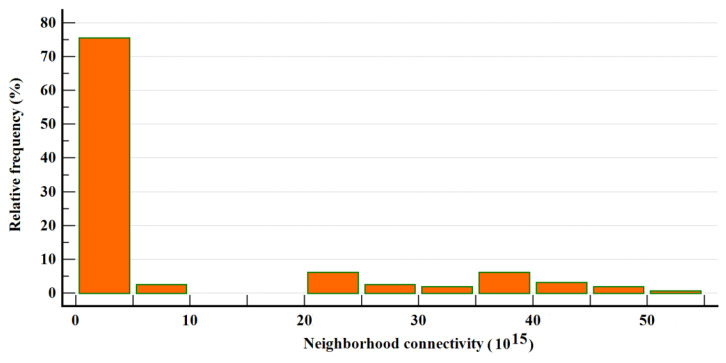
Distribution of neighborhood connectivity of the author network in the field of Paralympic powerlifting.

**Figure 5:**
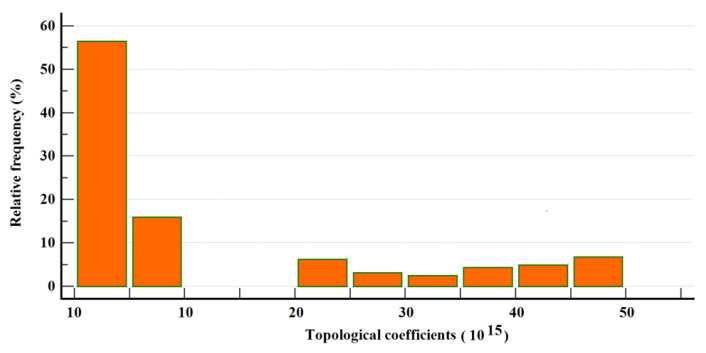
Distribution of topological coefficients of the author network in the field of Paralympic powerlifting.

Similar highly asymmetrical distributions can be found for other topological parameters, for instance, for neighborhood connectivity (Figure 4) and topological coefficients (Figure 5).

Finally, in terms of publication years, 13 articles (35.1%) were published in 2022.

## 4. Discussion

Despite having already become a paralympic sports discipline in 1984 [63], only in the last few years has PP been attracting a lot of interest from the community of researchers, with the first scholarly contribution dating back to 2012 and with more than one-third of the scientific output being published this year (2022). As such, the scholarly discipline is quite recent and young. Moreover, the community dealing with this topic is poorly interconnected, with most authors contributing to just one article, and with one single author being a hub node of the author network. Distributions of the number of articles and authors/co-authors were found to be highly asymmetrical, indicating that this research is still in its infancy and has great room as well as great potential to grow. Reflecting this, many research topics are also overlooked and underdeveloped, with the currently available evidence being based on a few studies.

### 4.1. Physiological and Psychological Responses to Warm-up, Training, Exercise, and Recovery in Paralympic Powerlifting 

Warm-ups have been acknowledged as instrumental in enhancing athletes’ performance-related outcomes in different sports disciplines and in a wide range of exercises and physical activities, by increasing body temperature and thus decreasing stiffness, improving nerve conduction velocity, and optimizing metabolic efficiency [64]. However, disabled people may have impaired thermoregulatory responses to exercise and could benefit less from warm-ups. In PP, the warm-up, regardless of its design and format (in terms of type, intensity, and volume), has been shown by two studies [26,27] to improve performance (type, intensity, volume), even though the body of evidence is limited (being based on two studies only), and more research should be conducted to explore the effects of other designs of warm-up strategies.

Concerning training, the use of elastic bands (vs. the fixed-resistance methodology) in PP seems to promote overload, increase fatigue, and decrease strength, whilst it appears to be an effective and common practice in powerlifting [65]. However, this conclusion is drawn from one study only [28], warranting further research in the field. As well, the determinants of training in terms of the optimal preparation interval should be further investigated: the only study available [29] failed to detect any age-, sex-, and gender-specific differences. 

In addition, disabled people have impaired cardiovascular, respiratory, neuromuscular, and thermoregulatory responses to exercise. However, regular training can compensate for these impairments and derangements [30,31], helping this specific population overcome their underlying limitations [47,48,49,50], reaching levels of relative and absolute strength sometimes greater than able-bodied athletes [55]. PP athletes experience unique stressors [32], even though, through anticipatory and proactive coping functions, self-consciousness, and strong positive beliefs, PP athletes can become confident, successful, and inspirational figures for other people with disabilities [33].

These results are in line with studies evaluating physiological responses in para-athletics athletes [66,67], providing further evidence that high-level and long-term training can overcome the difficulties associated with the disability [68].

Evidence drawn from the studies overviewed in the present review confirms that PP is a safe sports discipline that can be practiced by people with disabilities [51,52,53,54,55,56]. However, the data revealed a high rate of chronic overuse injuries of the upper limbs among PP athletes, with the highest injury incidence rate among all other sports, second only to 5-a-side football [69]. These injuries, if not treated promptly, could have functional consequences in everyday activities. 

Concerning recovery strategies, pharmacological interventions (such as ibuprofen) were found to impact the immune and muscular systems, proving to be an important recovery strategy to reduce fatigue and improve performance, albeit without any effect on oxidative and anti-inflammatory stress markers, as shown in the two studies by Aidar et al. [42,43], whilst the results were mixed in the study by Fraga et al. [44], in which ibuprofen seemed to delay the anti-inflammatory response post-exercise. Another study [45] investigated the impact of creatine supplementation on recovery, which was found to impact one parameter only, showing a decrease in fatigue index values. Finally, another study [46] explored different post-workout recovery strategies (namely, passive recovery, dry needling, and cold-water immersion), finding that recovery strategies differentially contributed in terms of outcomes measured and timing of the return to baseline values of altered homeostasis induced by the training session.

### 4.2. Methodological and Statistical Advancements 

Methodological and statistical advancements [57,58,59,60,61,62] have enabled a better assessment of performance outcomes, for which classical approaches relying on the null-hypothesis significant statistic may be misleading. A few studies have utilized the magnitude-based inference (MBI) model, which seems to be a promising and better statistical proposition compared with conventional inferential statistics to quantitatively analyze and interpret sports performance outcomes. FANOVA, as well, has been very recently introduced in the field of biomechanics and neuromechanics and has enabled the uncovering of specific patterns that classical methodologies failed to discover. Biomechanical studies conducted in PP failed to detect significant differences or found only slight differences in terms of the performance of PP under different BP conditions (such as with the legs tied and untied [34,35], using different grip widths [36,37], with the arched and flat techniques [38], at different distances from the bar to the chest [40], and in different sub-phases of the BP movement [41]). The use of novel, cutting-edge techniques could potentially result in the discovery of subtle differences. Moreover, studies on paralympic athletes could further benefit from big data analytics (BDA) and artificial intelligence (AI)-based techniques.

The 1RM test is the gold standard for defining and controlling athlete strength and can be used to track strength progress and calculate percentage loads. However, it is not a practical test, requires special attention, presents a high risk of injury, and could compromise the prescribed training session of the day. The study that examined the relationships between different loading intensities and movement velocities was able to accurately predict the maximum of one repetition. Furthermore, another practicable alternative for regular load monitoring was the RIR-based scale [62].

### 4.3. Gaps in Knowledge and Future Directions 

Moreover, we identified some gaps in the knowledge concerning PP: namely, (1) psychological, (2) nutritional aspects, and (3) doping. The latter topic is particularly paramount, in that doping substances and methods/practices, including the intentional activation of autonomic dysreflexia, also called “boosting” [70], are well known to be widespread among PP athletes and other para-athletes [22,71]. Furthermore, there is a significant lack of studies that have stratified data and outcomes based on the type, cause, and severity of the disability and how these clinical features affect sports and physical performance. For example, it is known that super-compensation processes and fatigue responses are different among Paralympic athletes, and tailor-made programming is required for correct and safe psychophysical preparation [72,73].

While kinematics assessments have been widely investigated, the methodology of training and periodization theory related to peak performance represent research areas relatively under-explored and overlooked in the extant scholarly literature. For example, most studies attribute changes in performance only to the effectiveness of short-term programs rather than long-term training programs. In addition, there are no follow-up studies. However, it is possible that a training methodology carried out over longer periods of time leads to longer-lasting physiological adaptations and that acquired gains in strength, velocity, and endurance may fade away with training cessation [74].

Besides, despite the increasing presence of female athletes in the discipline of PP, very few studies have recruited women. In these studies, it emerges that the woman para-athletes are less strong and powerful, but little information is present on the differences in response to exercise, on the recovery times, and on the correct parameters of the load to be used compared to equally trained men.

Finally, there is a tremendous paucity of information about how the still ongoing “Coronavirus Disease 2019” (COVID-19) pandemic has impacted PP. Information and recommendations and the highlighting of potential strategies and approaches would be crucial as it is possible to predict that new emerging/re-emerging infectious outbreaks can be anticipated to occur in the next future. 

Although there are studies that provide recommendations for endurance and team-sport para-athletes to maintain general and sport-specific conditioning [75,76,77], these methods of home-based training may not be valid for strength para-athletes due to the need to use specific pieces of bulky, heavy, and expensive equipment [78].

## Figures and Tables

**Table 1:** The ten most productive authors on Paralympic powerlifting.

Author	Country	Number of Items (%)	Number of Links	Total Link Strength	Author Cluster
Aidar, F.J.	Brazil	22 (59.5%)	94	264	7
Cabral, B.G.	Brazil	15 (40.5%)	76	209	2
de Almeida-Neto, P.F.	Brazil	15 (40.5%)	74	207	2
de Matos, D.G.	Brazil/Canada	14 (37.8%)	63	188	1
Marçal, A.C.	Brazil	13 (35.1%)	62	182	1
Reis, V.M.	Portugal	11 (29.7%)	56	150	1
Clemente, F.M.	Portugal	11 (29.7%)	54	148	6
de Souza, R.F.	Brazil	11 (29.7%)	54	145	1
Garrido, N.D.	Portugal	10 (27.0%)	52	145	3
dos Santos, J.L.	Brazil	7 (18.9%)	46	98	3

**Table 2:** The main topological features of the scholarly community of authors on Paralympic powerlifting.

Topological Feature	Value
Average number of neighbors	20.78
Network diameter	4
Network radius	2
Characteristic path length	1.91
Clustering coefficient	0.84
Network density	0.20
Network heterogeneity	0.79
Network centralization	0.73
Connected components	11

## Data Availability

Not applicable.

## References

[1] Ferland P.M., Comtois A.S. (2019). Classic Powerlifting Performance: A Systematic Review. J. Strength Cond. Res..

[2] Bengtsson V., Berglund L., Aasa U. (2018). Narrative review of injuries in powerlifting with special reference to their association to the squat, bench press and deadlift. BMJ Open Sport Exerc. Med..

[3] Travis S.K., Mujika I., Gentles J.A., Stone M.H., Bazyler C.D. (2020). Tapering and Peaking Maximal Strength for Powerlifting Performance: A Review. Sports.

[4] International Paralympic Committee (2020). World Para Powerlifting Technical Rules and Regulations.

[5] Mann D.L., Tweedy S.M., Jackson R.C., Vanlandewijck Y.C. (2021). Classifying the evidence for evidence-based classification in Paralympic sport. J. Sport. Sci..

[6] Grant M.J., Booth A. (2009). A typology of reviews: An analysis of 14 review types and associated methodologies. Health Info. Libr. J..

[7] Shi P., Bairner A. (2022). Sustainable Development of Olympic Sport Participation Legacy: A Scoping Review Based on the PAGER Framework. Sustainability.

[8] Munn Z., Pollock D., Price C., Aromataris E., Stern C., Stone J., Barker T.H., Godfrey C.M., Clyne B., Booth A. (2022). Investigating different typologies for the synthesis of evidence: A scoping review protocol. JBI Evid. Synth..

[9] Khalil H., Tricco A.C. (2022). Differentiating between mapping reviews and scoping reviews in the evidence synthesis ecosystem. J. Clin. Epidemiol..

[10] Arksey H., O’Malley L. (2005). Scoping studies: Towards a methodological framework. Int. J. Soc. Res. Methodol..

[11] Westphaln K.K., Regoeczi W., Masotya M., Vazquez-Westphaln B., Lounsbury K., McDavid L., Lee H., Johnson J., Ronis S.D. (2021). From Arksey and O’Malley and Beyond: Customizations to enhance a team-based, mixed approach to scoping review methodology. MethodsX.

[12] Bradbury-Jones C., Aveyard H., Herber O.R., Isham L., Taylor J., O’Malley L. (2021). Scoping reviews: The PAGER framework for improving the quality of reporting. Int. J. Soc. Res. Methodol..

[13] Mbogning Fonkou M.D., Bragazzi N.L., Tsinda E.K., Bouba Y., Mmbando G.S., Kong J.D. (2021). COVID-19 Pandemic Related Research in Africa: Bibliometric Analysis of Scholarly Output, Collaborations and Scientific Leadership. Int. J. Env. Res. Public Health.

[14] van Eck N.J., Waltman L. (2010). Software survey: VOSviewer, a computer program for bibliometric mapping. Scientometrics.

[15] Bastian M., Heymann S., Jacomy M. Gephi: An Open Source Software for Exploring and Manipulating Networks. Proceedings of the International AAAI Conference on Web and Social Media.

[16] Shannon P., Markiel A., Ozier O., Baliga N.S., Wang J.T., Ramage D., Amin N., Schwikowski B., Ideker T. (2003). Cytoscape: A software environment for integrated models of biomolecular interaction networks. Genome Res..

[17] Krnc M., Škrekovski R. (2020). Group Degree Centrality and Centralization in Networks. Mathematics.

[18] Jacob R., Harikrishnan K.P., Misra R., Ambika G. (2017). Measure for degree heterogeneity in complex networks and its application to recurrence network analysis. R. Soc. Open Sci..

[19] Blauwet C.A., Chakraverty J., Derman W., Idrisova G., Martin P., Miller S.C., Morrissey D., Webborn N. (2022). Shoulder Pain, Function, and Ultrasound-Determined Structure in Elite Wheelchair-Using Para Athletes: An Observational Study. Med. Sci. Sport. Exerc..

[20] Von Held R., Castilho T., Antunes L., Tavares J., Pivetta Petinati M.F., Winckler C., Neto Z., Scariot R., Küchler E.C., Brancher J.A. (2021). Interleukin 1 alpha genetic polymorphisms as potential biomarkers for oral health-related quality of life in Para athletes. Spec. Care Dent..

[21] Durán Agüero S., Arroyo Jofre P., Varas Standen C., Herrera-Valenzuela T., Moya Cantillana C., Pereira Robledo R., Valdés-Badilla P. (2015). Calidad Del Sueño, Somnolencia E Insomnio En Deportistas Paralímpicos De Elite Chilenos [Sleep Quality, Excessive Daytime Sleepiness And Insomnia In Chilean Paralympic Athletes]. Nutr. Hosp..

[22] Van de Vliet P. (2012). Antidoping in paralympic sport. Clin. J. Sport Med..

[23] Echague C.G., Csokmay J.M. (2018). Exercise-Induced Abdominal Wall Muscle Injury Resulting in Rhabdomyolysis and Mimicking an Acute Abdomen. Obstet. Gynecol..

[24] Silva A., Pinto Pinheiro L.S., Silva S., Andrade H., Pereira A.G., Rodrigues da Silva F., Guerreiro R., Barreto B., Resende R., Túlio de Mello M. (2022). Sleep in Paralympic athletes and its relationship with injuries and illnesses. Phys. Ther. Sport.

[25] Brancher J.A., Morodome F., Madalena I.R., Reis C., Von Held R., Antunes L., Winckler C., Salgueirosa F., Neto Z., Storrer C. (2021). Salivary pH and oral health of Brazilian para-athletes: Saliva and oral health of para-athletes. Spec. Care Dent..

[26] Resende M.A., Vasconcelos Resende R.B., Reis G.C., Barros L.O., Bezerra M., Matos D.G., Marçal A.C., Almeida-Neto P.F., Cabral B., Neiva H.P. (2020). The Influence of Warm-Up on Body Temperature and Strength Performance in Brazilian National-Level Paralympic Powerlifting Athletes. Medicina.

[27] de Aquino Resende M., Aidar F.J., Vasconcelos Resende R.B., Reis G.C., de Oliveira Barros L., de Matos D.G., Marçal A.C., de Almeida-Neto P.F., Díaz-de-Durana A.L., Merino-Fernández M. (2021). Are Strength Indicators and Skin Temperature Affected by the Type of Warm-Up in Paralympic Powerlifting Athletes?. Healthcare.

[28] Aidar F.J., Clemente F.M., de Lima L.F., de Matos D.G., Ferreira A., Marçal A.C., Moreira O.C., Bulhões-Correia A., de Almeida-Neto P.F., Díaz-de-Durana A.L. (2021). Evaluation of Training with Elastic Bands on Strength and Fatigue Indicators in Paralympic Powerlifting. Sports.

[29] Lopes-Silva J.P., Richardson D., Fukuda D.H., Franchini E. (2021). Is there an optimal interval for medal winning performance in World Para Powerlifting competition?. Am. J. Phys. Med. Rehabil..

[30] Paz Â.A., Aidar F.J., de Matos D.G., de Souza R.F., da Silva-Grigoletto M.E., van den Tillaar R., Ramirez-Campillo R., Nakamura F.Y., Costa M., Nunes-Silva A. (2020). Comparison of Post-Exercise Hypotension Responses in Paralympic Powerlifting Athletes after Completing Two Bench Press Training Intensities. Medicina.

[31] Aidar F.J., Paz Â.A., Gama D.M., de Souza R.F., Vieira Souza L.M., Santos J., Almeida-Neto P.F., Marçal A.C., Neves E.B., Moreira O.C. (2021). Evaluation of the Post-Training Hypotensor Effect in Paralympic and Conventional Powerlifting. J. Funct. Morphol. Kinesiol..

[32] Silva D., Santos M., Aidar F., Cabral B., Stieler E., Resende R., Andrade A., Almeida-Neto P., Bulhões-Correia A., Guerreiro R. (2022). Effect of strength training on psychophysiological aspects in Paralympic powerlifting athletes: A pilot study. Hum. Mov..

[33] Roy A. (2012). Socio-cultural power dynamics and coping functions: A narrative case report of a female paralympian. Asian, J. Sport. Med..

[34] Guerra I., Aidar F.J., Greco G., de Almeida-Neto P.F., De Candia M., de Araújo Tinoco Cabral B.G., Poli L., Filho M.M., Carvutto R., Silva A.F. (2022). Are sEMG, Velocity and Power Influenced by Athletes’ Fixation in Paralympic Powerlifting?. Int. J. Environ. Res. Public Health.

[35] Mota M.G., Aidar F.J., Rocha J.C.S.D., Santos W.D.S., Leite Junior J.A.D.S., Jesus J.B.D., Freitas A.V.D., Solidade V.T.D. (2020). Avaliação de duas formas de execução, amarrado ou não no powerlifting paralímpico: Um estudo piloto. Motricidade.

[36] Dos Santos M., Aidar F.J., Alejo A.A., de Matos D.G., de Souza R.F., de Almeida-Neto P.F., de Araújo Tinoco Cabral B.G., Nikolaidis P.T., Knechtle B., Clemente F.M. (2021). Analysis of Grip Amplitude on Velocity in Paralympic Powerlifting. J. Funct. Morphol. Kinesiol..

[37] Dos Santos M., Aidar F.J., de Souza R.F., Dos Santos J.L., da Silva de Mello A., Neiva H.P., Marinho D.A., Marques M.C. (2020). Does the Grip Width Affect the Bench Press Performance of Paralympic Powerlifters?. Int. J. Sport. Physiol. Perform..

[38] Ribeiro Neto F., Dorneles J.R., Luna R.M., Spina M.A., Gonçalves C.W., Gomes Costa R.R. (2022). Performance Differences Between the Arched and Flat Bench Press in Beginner and Experienced Paralympic Powerlifters. J. Strength Cond. Res..

[39] Mendonça T.P., Aidar F.J., Matos D.G., Souza R.F., Marçal A.C., Almeida-Neto P.F., Cabral B.G., Garrido N.D., Neiva H.P., Marinho D.A. (2021). Force production and muscle activation during partial vs. full range of motion in Paralympic Powerlifting. PLoS ONE.

[40] Aidar F.J., Clemente F.M., Matos D.G., Marçal A.C., de Souza R.F., Moreira O.C., Almeida-Neto P.F., Vilaça-Alves J., Garrido N.D., Dos Santos J.L. (2021). Evaluation of Strength and Muscle Activation Indicators in Sticking Point Region of National-Level Paralympic Powerlifting Athletes. J. Funct. Morphol. Kinesiol..

[41] da Silva B.G., Miziara I.M., Furtado D.A., dos Santos S.S., Fidale T.M., Pereira A.A. (2022). Electromyographical activity of the pectoralis, triceps, and deltoideus during the sub-phases of bench press in paralympic powerlifters. Sport. Eng..

[42] Aidar F.J., Fraga G.S., Getirana-Mota M., Marçal A.C., Santos J.L., de Souza R.F., Ferreira A., Neves E.B., Zanona A.F., Bulhões-Correia A. (2021). Effects of Ibuprofen Use on Lymphocyte Count and Oxidative Stress in Elite Paralympic Powerlifting. Biology.

[43] Aidar F.J., Fraga G.S., Getirana-Mota M., Marçal A.C., Santos J.L., de Souza R.F., Vieira-Souza L.M., Ferreira A., de Matos D.G., de Almeida-Neto P.F. (2022). Evaluation of Ibuprofen Use on the Immune System Indicators and Force in Disabled Paralympic Powerlifters of Different Sport Levels. Healthcare.

[44] Fraga G.S., Aidar F.J., Matos D.G., Marçal A.C., Santos J.L., Souza R.F., Carneiro A.L., Vasconcelos A.B., Da Silva-Grigoletto M.E., van den Tillaar R. (2020). Effects of Ibuprofen Intake in Muscle Damage, Body Temperature and Muscle Power in Paralympic Powerlifting Athletes. Int. J. Environ. Res. Public Health.

[45] Soares Freitas Sampaio C.R., Aidar F.J., Ferreira A., Santos J., Marçal A.C., Matos D.G., Souza R.F., Moreira O.C., Guerra I., Fernandes Filho J. (2020). Can Creatine Supplementation Interfere with Muscle Strength and Fatigue in Brazilian National Level Paralympic Powerlifting?. Nutrients.

[46] Santos W., Aidar F.J., Matos D.G., Van den Tillaar R., Marçal A.C., Lobo L.F., Marcucci-Barbosa L.S., Machado S., Almeida-Neto P.F., Garrido N.D. (2021). Physiological and Biochemical Evaluation of Different Types of Recovery in National Level Paralympic Powerlifting. Int. J. Environ. Res. Public Health.

[47] Gołaś A., Zwierzchowska A., Maszczyk A., Wilk M., Stastny P., Zając A. (2017). Neuromuscular Control During the Bench Press Movement in an Elite Disabled and Able-Bodied Athlete. J. Hum. kinetics.

[48] Szafraniec R., Kisilewicz A., Kumorek M., Kristiansen M., Madeleine P., Mroczek D. (2020). Effects of High-Velocity Strength Training on Movement Velocity and Strength Endurance in Experienced Powerlifters with Cerebral Palsy. J. Hum. Kinet..

[49] Teles L., Aidar F.J., Matos D.G., Marçal A.C., Almeida-Neto P.F., Neves E.B., Moreira O.C., Ribeiro Neto F., Garrido N.D., Vilaça-Alves J. (2021). Static and Dynamic Strength Indicators in Paralympic Power-Lifters with and without Spinal Cord Injury. Int. J. Environ. Res. Public Health.

[50] Aidar F.J., Cataldi S., Badicu G., Silva A.F., Clemente F.M., Latino F., Greco G., Fischetti F. (2022). Paralympic Powerlifting as a Sustainable Way to Improve Strength in Athletes with Spinal Cord Injury and Other Disabilities. Sustainability.

[51] Lopes-Silva J.P., Richardson D., Franchini E. (2022). Chronological Age and Performance in Paralympic Powerlifters: Differences Between Sexes, Competition, and Weight Categories. J. Sci. Sport Exerc..

[52] Willick S.E., Cushman D.M., Blauwet C.A., Emery C., Webborn N., Derman W., Schwellnus M., Stomphorst J., Van de Vliet P. (2016). The epidemiology of injuries in powerlifting at the London 2012 Paralympic Games: An analysis of 1411 athlete-days. Scand. J. Med. Sci. Sport..

[53] Jarraya M., Blauwet C.A., Crema M.D., Heiss R., Roemer F.W., Hayashi D., Derman W.E., Guermazi A. (2021). Sports injuries at the Rio de Janeiro 2016 Summer Paralympic Games: Use of diagnostic imaging services. Eur. Radiol..

[54] AHamid M.S., Ghazali S.S., Karim S.A. (2019). Anthropometric Characteristics of Malaysian Competitive Powerlifters with Physical Disabilities. J. Health Transl. Med..

[55] van den Hoek D., Garrett J., Howells R., Latella C. (2022). Paralympians Are Stronger Than You Know: A Comparison of Para and Nondisabled Powerlifting Bench Press World Records. J. Strength Cond. Res..

[56] Severin A.C., Baumgart J.K., Haugen T., Hogarth L. (2022). Peak age and performance trajectories in Para powerlifters. Am. J. Phys. Med. Rehabil..

[57] Ramos Dalla Bernardina G., Danillo Matos Dos Santos M., Alves Resende R., Túlio de Mello M., Rodrigues Albuquerque M., Augusto Paolucci L., Carpes F.P., Silva A., Gustavo Pereira de Andrade A. (2021). Asymmetric velocity profiles in Paralympic powerlifters performing at different exercise intensities are detected by functional data analysis. J. Biomech..

[58] Bellitto A., Marchesi G., Comini M., Massone A., Casadio M., De Luca A. (2022). Electromyographic and Kinematic Evaluation of Bench Press Exercise: A Case Report Study on Athletes with Different Impairments and Expertise. Sport Sci. Health.

[59] Loturco I., Pereira L.A., Winckler C., Santos W.L., Kobal R., McGuigan M. (2019). Load-Velocity Relationship in National Paralympic Powerlifters: A Case Study. Int. J. Sport. Physiol. Perform..

[60] Aidar F.J., Brito C.J., de Matos D.G., de Oliveira L., de Souza R.F., de Almeida-Neto P.F., de Araújo Tinoco Cabral B.G., Neiva H.P., Neto F.R., Reis V.M. (2022). Force-velocity relationship in Paralympic powerlifting: Two or multiple-point methods to determine a maximum repetition. BMC Sport. Sci. Med. Rehabil..

[61] Aidar F.J., Cataldi S., Badicu G., Silva A.F., Clemente F.M., Bonavolontà V., Greco G., Getirana-Mota M., Fischetti F. (2022). Does the Level of Training Interfere with the Sustainability of Static and Dynamic Strength in Paralympic Powerlifting Athletes?. Sustainability.

[62] Neto F.R., Dorneles J.R., Aidar F.J., Gonçalves C.W., Veloso J., Costa R. (2022). Validation of the Repetitions in Reserve Rating Scale in Paralympic Powerlifting Athletes. Int. J. Sport. Med..

[63] World Para Powerlifting. History of para powerlifting. https://www.paralympic.org/powerlifting/about.

[64] Ribeiro B., Pereira A., Neves P.P., Sousa A.C., Ferraz R., Marques M.C., Marinho D.A., Neiva H.P. (2020). The Role of Specific Warm-up during Bench Press and Squat Exercises: A Novel Approach. Int. J. Environ. Res. Public Health.

[65] Shaw M.P., Andersen V., Sæterbakken A.H., Paulsen G., Samnøy L.E., Solstad T. (2022). Contemporary Training Practices of Norwegian Powerlifters. J. Strength Cond. Res..

[66] Runciman P., Derman W., Ferreira S., Albertus-Kajee Y., Tucker R. (2015). A descriptive comparison of sprint cycling performance and neuromuscular characteristics in able-bodied athletes and paralympic athletes with cerebral palsy. Am. J. Phys. Med. Rehabil..

[67] Runciman P., Tucker R., Ferreira S., Albertus-Kajee Y., Derman W.A. (2016). Effects of induced volitional fatigue on sprint and jump performance in paralympic athletes with cerebral palsy. Am. J. Phys. Med. Rehabil..

[68] Puce L., Pallecchi I., Chamari K., Marinelli L., Innocenti T., Pedrini R., Mori L., Trompetto C. (2021). Systematic Review of Fatigue in Individuals With Cerebral Palsy. Front. Hum. Neurosci..

[69] Willick S.E., Webborn N., Emery C., Blauwet C.A., Pit-Grosheide P., Stomphorst J., Van de Vliet P., Patino Marques N.A., Martinez-Ferrer J.O., Jordaan E. (2013). The epidemiology of injuries at the London 2012 Paralympic Games. Br. J. Sport. Med..

[70] Mazzeo F., Santamaria S., Iavarone A. (2015). “Boosting” in Paralympic athletes with spinal cord injury: Doping without drugs. Funct. Neurol..

[71] Walter M., Krassioukov A.V. (2018). Autonomic Nervous System in Paralympic Athletes with Spinal Cord Injury. Phys. Med. Rehabil. Clin. North Am..

[72] Puce L., Marinelli L., Pierantozzi E., Mori L., Pallecchi I., Bonifazi M., Bove M., Franchini E., Trompetto C. (2018). Training methods and analysis of races of a top level Paralympic swimming athlete. J. Exerc. Rehabil..

[73] Puce L., Bragazzi N.L., Currà A., Marinelli L., Mori L., Cotellessa F., Chamari K., Ponzano M., Samanipour M.H., Nikolaidis P.T. (2022). Not all Forms of Muscle Hypertonia Worsen With Fatigue: A Pilot Study in Para Swimmers. Front. Physiol..

[74] Hill K.G., Woodward D., Woelfel T., Hawkins J.D., Green S. (2016). Planning for Long-Term Follow-Up: Strategies Learned from Longitudinal Studies. Prev. Sci. Off. J. Soc. Prev. Res..

[75] Shaw K.A., Bertrand L., Deprez D., Ko J., Zello G.A., Chilibeck P.D. (2021). The impact of the COVID-19 pandemic on diet, fitness, and sedentary behaviour of elite para-athletes. Disabil. Health J..

[76] Cavaggioni L., Rossi A., Tosin M., Scurati R., Michielon G., Alberti G., Merati G., Formenti D., Trecroci A. (2022). Changes in Upper-Body Muscular Strength and Power in Paralympic Swimmers: Effects of Training Confinement during the COVID-19 Pandemic. Int. J. Environ. Res. Public Health.

[77] Peña-González I., Sarabia J.M., Manresa-Rocamora A., Moya-Ramón M. (2022). International football players with cerebral palsy maintained their physical fitness after a self-training program during the COVID-19 lockdown. PeerJ.

[78] Latella C., Haff G.G. (2020). Global Challenges of Being a Strength Athlete during a Pandemic: Impacts and Sports-Specific Training Considerations and Recommendations. Sports.

